# Primulina hochiensis var. ochroleuca (Gesneriaceae), a new variety from a limestone area of Guangxi, China, and errata on five new species of *Primulina*

**DOI:** 10.3897/phytokeys.152.50968

**Published:** 2020-07-07

**Authors:** Yu-Zhen Ge, Zi-Bing Xin, Long-Fei Fu, Wei-Chuen Chou, Yi Huang, Zhang-Jie Huang, Stephen Maciejewski, Fang Wen

**Affiliations:** 1 Guangxi Key Laboratory of Plant Conservation and Restoration Ecology in Karst Terrain, Guangxi Institute of Botany, Guangxi Zhuang Autonomous Region and Chinese Academy of Sciences, CN-541006 Guilin, China; 2 Gesneriad Conservation Center of China, Guilin Botanical Garden, Guangxi Zhuang Autonomous Region and Chinese Academy of Sciences, CN-541006 Guilin, China; 3 The Gesneriad Society, 1122 East Pike Street, PMB 637 Seattle, WA 98122-3916 USA

**Keywords:** Cliff-dwelling, flora of Guangxi, limestone flora, taxonomy

## Abstract

Primulina hochiensis var. ochroleuca, a new variety from a limestone hill of karst areas, Guangxi, China is described with color photographs. It resembles P. hochiensis var. hochiensis, P. hochiensis var. ovata and P. hochiensis var. rosulata, but can be easily distinguished by a combination of characteristics, especially by its corolla color. We found only one population with approx. 3000 mature individuals at the type locality. This variety is provisionally assessed as vulnerable [VU C1] using IUCN criteria.

## Introduction

By the end of December 2019, the genus *Primulina*Hance (1883) comprised over 220 species names (infraspecific taxa included) (Wen et al. 2019, 2020; IPNI 2020; Tropicos 2020), including many new species published in recent years (e.g., Pan et al. 2020). In all taxa of *Primulina* in the world at present, 208 were recorded from China and 21 were recorded from Vietnam (Vu 2018; Wen et al. 2020). As the largest genus of Gesneriaceae in China, *Primulina* s. l. has become representative of the rich diversity in the Chinese Gesneriaceae. Nevertheless, it is still possible to dig deeper into the biodiversity of *Primulina* (Möller 2019). Those highly diverse taxa mainly grow in limestone areas, which are highly fragmented and heterogeneous (Möller et al. 2016). Most species are micro-endemics with narrow, island-like distributions, often limited to a single cave or karst limestone hill system (Kang et al. 2014). This edaphic complexity may be a strong driver of speciation via habitat specialization (local adaptations) to edaphic microhabitats (Hao et al. 2015). In addition, Kong et al. (2017) suggest that global temperature change is probably the primary driver of diversification in *Primulina*. And the monsoons and edaphic characteristics are probably also strongly linked to its diversification.

*Primulina hochiensis* was first published as *Chirita hochiensis* C.C. Huang & X.X. Chen (1992). According to the results of molecular phylogenetic studies, almost all species of Chirita sect. Gibbosaccus C.B. Clarke, 1883 were merged into *Primulina* Hance (Wang et al. 2011; Weber et al. 2011), including *C. hochiensis*, which was revised as *P. hochiensis* (C.C. Huang & X.X. Chen) Mich. Möller and A. Weber. Primulina hochiensis var. rosulata F. Wen & Y.G. Wei from Guangxi, China was published as a variety (Wen et al. 2012), and was raised to the rank of species based on its phylogenetic distance from *P. hochiensis* and *P. yingdeensis* Z.L. Ning, M. Kang & X.Y. Zhuang (Ning et al. 2016), but was demoted again as a variety, after performing further population genetical analyses (Yang 2018; Yang et al. 2019). Meanwhile, *P. tsoongii* H.L. Liang, Bo Zhao & Fang Wen (Liang et al. 2013) was treated as a synonym of P. hochiensis var. rosulata, and another new variety, P. hochiensis var. ovata L.H. Yang, H.H. Kong & M. Kang, was confirmed and published (Yang et al. 2019).

Two amateurs of Gesneriaceae from Guangxi found this unknown taxon in the wild in late September 2017. The population was not in flowering at that time, only the white buds that were about to bloom. They thought it might be a member of *P. hochiensis* complex because its habit resembles P. hochiensis var. hochiensis and P. hochiensis var. rosulata, but differs from the former by its stolon absent, and from the latter by its conspicuously larger leaf blade and longer pedicel. Although it was thought to be *P. hochiensis*, some individuals were collected for cultivation. When all the individuals are in flower, they found that all the flowers are yellow and the color is very stable, hence it can be distinguished from all the other varieties by this character. They visited the original locality again in late October 2017 and found all the individuals’ flowers are yellow. Some living plants were collected and mailed to GCCC for further study. We grew them in common garden of GCCC with other varieties of *P. hochiensis* for two years and found that all the flowers of this unknown taxon are yellow, and can be distinguished from all the other varieties. And we made an extensive survey of the surrounding hills in October 2019 when this unknown taxon was in flower. No individual of this unknown taxon was found in the surrounding limestone hills, only some P. hochiensis var. ovata growing on those hills. Though the distribution of this new variety is close to P. hochiensis var. ovata, we can easily tell them apart.

After analyzing the morphological characters on these plants, and comparing them with the other three similar-looking *P. hochiensis* varieties, we confirmed that it is indeed a new variety of *P. hochiensis*. Thus, we describe it here.

## Taxonomic treatment

### Primulina hochiensis

Taxon classificationPlantaeLamialesGesneriaceae

(C.C.Huang & X.X.Chen) Mich.Möller & A.Weber var. ochroleuca, F.Wen Y.Z.Ge & Z.B.Xin var. nov.

C79BD987-1A98-5A2D-908E-EE29A537415E

urn:lsid:ipni.org:names:77210102-1

Figs 1 , 2A

#### Diagnosis.

The new variety can be easily distinguished from all varieties of *Primulina hochiensis* by its pale yellow corolla. It differs from the typical variety, P. hochiensis var. hochiensis by its stolon lacking and obviously longer petiole (5–7 cm long); from P. hochiensis var. ovata by its stolon lacking, obviously longer petiole (5–7 cm long) and longer pedicel (1.5–2 cm long); from P. hochiensis var. rosulata by its longer pedicel (1.5–2 cm long), shorter calyx (3.5–4 mm long), corolla throat with one big yellow patch and longer pistil (1.4–1.8 cm long).

**Figure 1.**
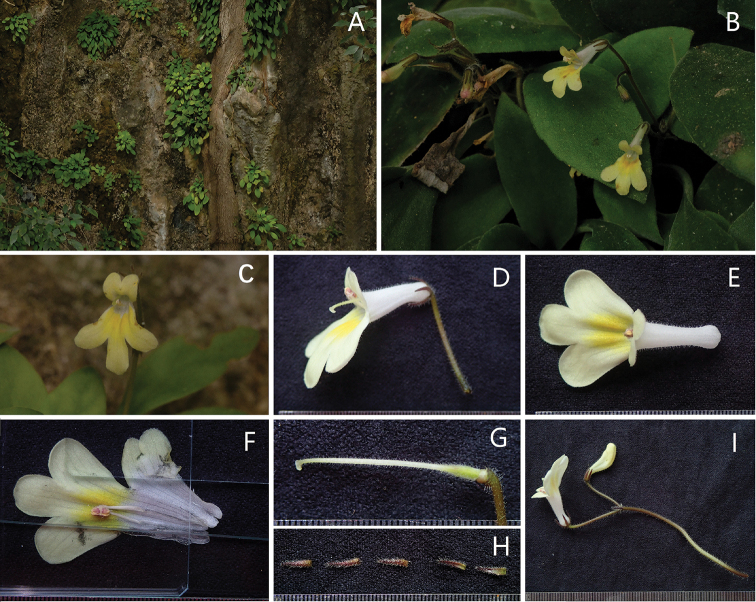
Primulina hochiensis var. ochroleuca**A** habitat **B** habit **C** frontal view of corolla **D** lateral view of corolla **E** top view of corolla **F** opened corolla with stamens and staminodes **G** pistil **H** calyx lobes **I** cyme with flowers (Photographed by Fang Wen).

#### Type.

China. Guangxi Zhuang Autonomous Region, Guilin City, Gongcheng County, Xiling Town, 24°55'N, 110°45'E, altitude ca. 220 m, 8 October 2019, *Fang Wen et al.*, *WF191008-03* (Holotype: IBK!; Isotypes: IBK!).

#### Description.

Herbs perennial, acaulescent. Leaves basal, 20–35; petiole cylindrical, densely extremely short pubescent, 5–7 × 0.4–0.5 cm; leaf blade elliptical to slightly ovate, 5.5–7.5 (–9) × 3–5 cm, densely appressed puberulent, base cuneate, margin entire, apex acute; lateral veins 4–6 on each side of the midrib, conspicuous on the abaxial surface, inconspicuous on the adaxial surface. Cymes 4–6, axillary, 1–3-branched, 2–8-flowered; peduncle 5–10 cm long, 1–1.5 mm in diameter, densely erect puberulent; bracts 2, opposite, linear, 3–3.5 × 1 mm, puberulent. Pedicel 1.5–2 cm long, 1–1.5 mm in diameter, puberulent. Calyx 5-parted from the base; segments equal, lanceolate-linear, 3.5–4 × 1–1.2 mm, densely pubescent, margin entire, apex acute. Corolla pale yellow, throat with two distinctly elliptic yellow spots, 2.5–3 cm long, orifice 0.6–0.8 cm in diameter, outside puberulent with both glandular and eglandular hairs, inside glabrous; tube narrowly infundibuliform, 1–1.2 cm long; limb distinctly 2-lipped, adaxial lip 2-parted to the base, lobes slightly oblique linguiform or ovate, ca. 5 × 2.5 mm; abaxial lip 3-parted to the middle, lobes obliquely ovate, ca. 8 × 4 mm. Stamens 2, adnate to ca 1.0 cm above the corolla base; anthers purple, reniform, ca. 1.5 × 1.2 mm, slightly constricted at the middle; filaments geniculate close to the base, ca. 6 mm long, glabrous; staminodes 3, lateral ones short linear, glabrous, 1–1.2 mm long, adnate to 4–5 mm above the corolla base, the central one linear, 0.8–1 mm long, adnate to 2.5–3 mm above the corolla base. Disc annular, margin entire or sometimes slightly erose, ca. 0.7 mm high. Pistil 1.4–1.8 cm long; ovary linear, 3–4 mm long, 1–1.5 mm in diameter, densely puberulent with both glandular and eglandular hairs; style 1.1–1.4 cm long, ca. 0.5 mm in diameter, glandular-puberulent. Stigmas translucent to white, obtrapeziform, apex 2-parted up to the middle, 0.8–1 mm long. Capsule linear, 1.8–2 cm long, ca. 1.5 mm in diameter, puberulent with both glandular and eglandular hairs.

**Figure 2.**
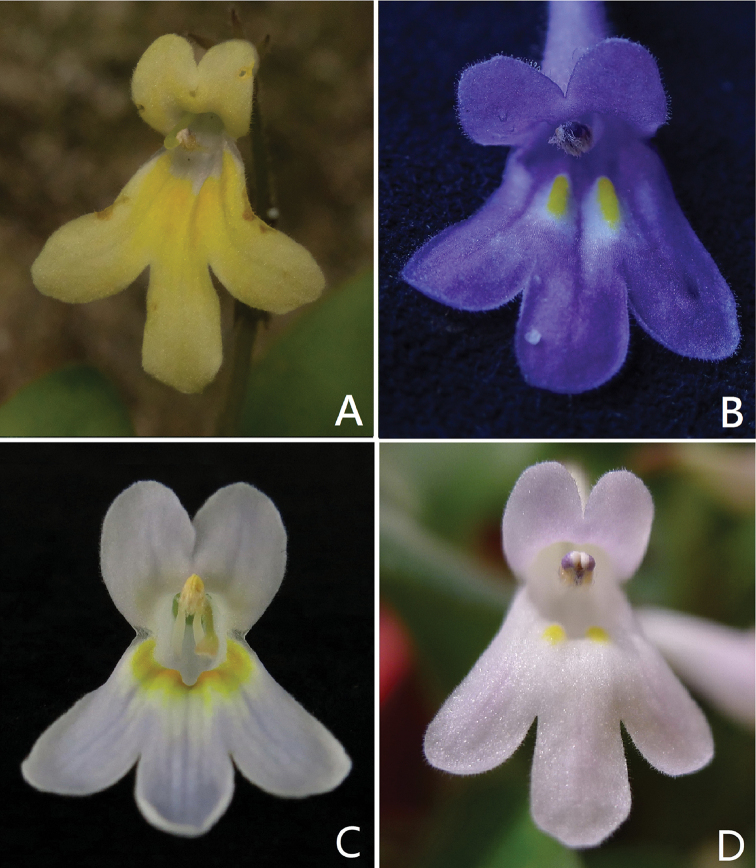
Comparison of frontal view of corolla between Primulina hochiensis var. ochroleuca and the most closely related taxa **A**P. hochiensis var. ochroleuca**B**P. hochiensis var. hochiensis**C**P. hochiensis var. ovata**D**P. hochiensis var. rosulata (**A, B, D** Photographed by Fang Wen; **C** Photographed by Li-Hua Yang).

#### Phenology.

Flowering occurs from September to November, and fruiting from November to January of the next year.

#### Etymology.

The specific epithet ‘*ochroleuca*’ is derived from its pale yellow corolla. The original epithet ‘*ochro*-*leuca*’ derived from the Greek, ‘ώχρα,’ namely ‘*ochra*-,’ means ochre, yellowish and ‘λευκά,’ namely ‘-*lefka*’ means white.

#### Vernacular name.

Huáng Huā Hé Chí Bào Chūn Jù Tái (Chinese pronunciation); 黄花河池报春苣苔 (Chinese name).

#### Distribution and habitat.

**Figure 3.**
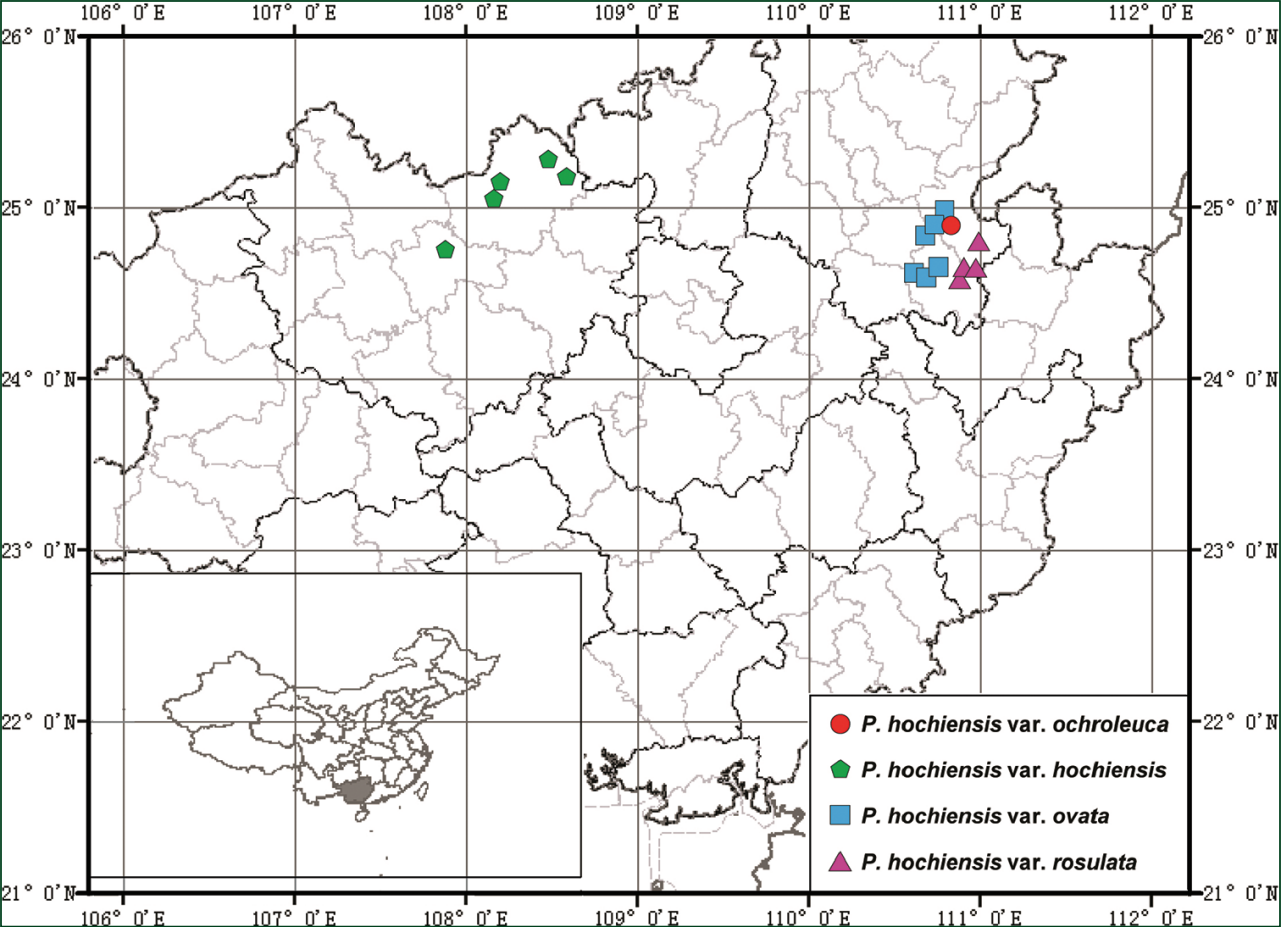
Geographical distribution of the Primulina hochiensis var. ochroleuca and the most closely related taxa.

Primulina hochiensis var. ochroleuca is hitherto only known from the type locality, Xiling Town, Gongcheng County, Guangxi Zhuang Autonomous Region, South China (Fig. 3), and grows on moist and shaded rocky surfaces on the cliff in subtropical evergreen seasonal rain forest.

#### Preliminary Conservation status.

The type population consists of approx. 3000 mature individuals, all growing on moist and shaded rocky surfaces on the cliff. They are easily disturbed by human activities because the distance from the type locality to the local village is short. Parts of vegetation of the type hill have been cleared by local people for fruit trees cultivation. Thus, following the IUCN Red List Categories and Criteria (IUCN 2019), it is temporarily assessed as vulnerable [VU C1].

#### Additional specimens examined.

***Primulina hochiensis*** (C.C. Huang & X.X. Chen) Mich. Möller & A. Weber var. ***hochiensis***, **China**: Guangxi, Hechi City, 23 October 1991, *C.C. Huang 19670* (Holotype: GXMI!); Huanjiang County, Shuiyuan Town to Xianan Town, limestone hill, 24°49'34.25"N, 108°01'59.01"E, 249 m, 19 Jul. 2013, *451226130719009LY* (GXMG!; IBK!). ***Primulina hochiensis*** (C.C. Huang & X.X. Chen) Mich. Möller & A. Weber var. ***ovata*** L.H. Yang, H.H. Kong & M. Kang, **China**: Guangxi, Guilin City, Pingle County, Pingle Town, Mawei Village, grows on moist limestone rocks at a lower elevation (150–300 m), 18 June 2016, *L.H. Yang PLMW* (holotype: IBSC!); Pingle County, Ertang Town, Da’e’shan Village, 18 June 2016, *L.H. Yang PLET* (IBSC!); Pingle County, Shazi Town, Bao’an Village, 20 June 2016, *L.H. Yang PLSZ* (IBSC!); Pingle County, Pingle Town, Taiping Village, 6 July 2016, *L.H. Yang and M. Kang PLMW* (IBSC!); Gongcheng County, Xiling Town, Huzimiao Village, 19 June 2016, *L.H. Yang GCXL01* (IBSC!); Gongcheng County, Xiling Town, Panyan Village, 19 June 2016, *L.H. Yang GCXL02* (IBSC!). ***Primulina hochiensis*** (C.C. Huang & X.X. Chen) Mich. Möller & A. Weber var. ***rosulata*** F. Wen & Y.G. Wei, **China**: Guangxi, Guilin City, Pingle County, Tong’an Town, growing in the entrance of a limestone cave, 24°34’47"N, 110°55'34"E, elevation ca. 149 m, 17 August 2008 (fl.), *B. Gao 08171* (holotype IBK!; isotype BJFC!); Gongcheng County, Lianhua Town, on moist limestone rock faces in evergreen broadleaved forest and bushes, located in the subtropical monsoon region, 161 m a.s.l., 11 Jul 2012, *Hui-Ling Liang*, *Yan-Cai Shi & De-Xin Kong*, *120711* (IBK!).

#### Notes.

The morphological comparisons between P. hochiensis var. ochroleuca and the most closely related taxa (P. hochiensis var. hochiensis, P. hochiensis var. ovata and P. hochiensis var. rosulata) are provided in Table 1.

**Table 1.** Morphological comparisons of Primulina hochiensis var. ochroleuca and the most closely related taxa.

Characters	P. hochiensis var. ochroleuca	P. hochiensis var. hochiensis	P. hochiensis var. ovata	P. hochiensis var. rosulata
**Stolon**	lacking	**conspicuous**	**conspicuous**	lacking
**Size of petiole**	5–7 × 0.4–0.5 cm	**1–3.5 × ca. 0.3 cm**	**2–4.5 × 0.2–0.4 cm**	3.0–5.5 × 0.2–0.4 cm
**Length of pedicel**	1.5–2 cm	0.7–2.3 cm	**0.8–1.2 cm**	≤ **0.7 cm**
**Size of calyx**	3.5–4 × 1–1.2 mm	4–7 × 0.5–0.8 mm	**4.5–6 × 1–1.5 mm**	**7–7.5 × 1.5–2.3 mm**
**Color of corolla**	pale yellow	**dark purple**	**pale purple or white**	**white or pale pink**
**Throat**	1 big patch	**2 small spots**	1 big patch	**2 small spots**
**No. of staminodes**	3	**2**	3	3
**Length of pistil**	14–18 mm	15–20 mm	16–18 mm	**9.2–9.7 mm**

**Note**: The bold words mean the key differences between each variety and the new one.

### Key to the varieties of the *Primulina hochiensis* complex

**Table:** 

1	Stolon conspicuous	**2**
–	Stolon lacking	**3**
2	With 2 small spots at throat of the corolla	**1. P. hochiensis var. hochiensis**
–	With 1 big patch at throat of the corolla	**2. P. hochiensis var. ovata**
3	With 2 small spots at throat of the corolla	**3. P. hochiensis var. rosulata**
–	With 1 big patch at throat of the corolla	**4. P. hochiensis var. ochroleuca**

## 

In Li et al. (2019), five new species belonging to the genus *Primulina* were described. The correct collection dates and the type specimens numbers of these are as follows:

Page 79, ***Primulina purpureokylin* F. Wen, Yi Huang & W. Chuen Chou**

The correct collection date of the type specimens of *Primulina purpureokylin* is 16 Nov 2017, not 3 Apr 2018.

Page 81, ***Primulina persica* F. Wen, Yi Huang & W. Chuen Chou**

The correct collection date and the number of the type specimens of *Primulina persica* is 25 Apr 2017, *Chou Wei Chuen et al. CWC170425-01*.

Page 83 ***Primulina cerina* F. Wen, Yi Huang & W. Chuen Chou**

The correct collection date and the number of the type specimens of *Primulina cerina* is 14 Apr 2017, *Chou Wei Chuen et al. CWC170414-01*.

Page 85 ***Primulina niveolanosa* F. Wen, S. Li & W. Chuen Chou**

The correct collection date and the number of the type specimens of *Primulina niveolanosa* are 8 Jun 2017, *Chou Wei Chuen et al. CWC170608-01*.

Page 87 ***Primulina leiyyi* F. Wen, Z. B. Xin & W. Chuen Chou**

The correct collection date of the type specimens of *Primulina leiyyi* is 8 Dec 2018, not 3 Apr 2018.

## Supplementary Material

XML Treatment for Primulina hochiensis

## References

[B1] Hance HF (1883) New Chinese Cyrtandreae. Le Journal de Botanique 21: 165–170.

[B2] Hao Z Kuang YW Kang M (2015) Untangling the influence of phylogeny, soil and climate on leaf element concentrations in a biodiversity hotspot. Functional Ecology 59(2): 165–176. 10.1111/1365-2435.12344

[B3] Huang CC Chen XX (1992) A new medicinal species of *Chirita* (Gesneriaceae) from Guangxi. Botanical Journal of South China 1: 14–16.

[B4] IPNI (2020) The International Plant Names Index. http://www.ipni.org [accessed May 2020]

[B5] IUCN (2019) Guidelines for Using the IUCN Red List Categories and Criteria. Version 14. Prepared by the Standards and Petitions Subcommittee of the IUCN Species Survival Commission. http://cmsdocs.s3.amazonaws.com/RedListGuidelines.pdf

[B6] Kang M Tao J Wang J Ren C Qi Q Xiang QY Huang HW (2014) Adaptive and nonadaptive genome size evolution in karst endemic flora of China. The New Phytologist 202(4): 1371–1381. 10.1111/nph.12726 24533910

[B7] Kong HH Condamine FL Harris AJ Chen JL Pan B Möller M Hoang VS Kang M (2017) Both temperature fluctuations and East Asian monsoons have driven plant diversification in the karst ecosystems from southern China. Molecular Ecology 26(22): 6414–6429. 10.1111/mec.14367 28960701

[B8] Li S Xin ZB Chou WC Huang Y Pan B Maciejewski S Wen F (2019) Five new species of the genus *Primulina* (Gesneriaceae) from limestone areas of Guangxi Zhuangzu autonomous region, China. PhytoKeys 127: 77–91. 10.3897/phytokeys.127.35445 31379451 PMC6661265

[B9] Liang HL Kong DX Shi YC Zhao B Wen F (2013) *Primulina tsoongii* sp. nov. (Gesneriaceae) from a limestone area in north Guangxi, China. Nordic Journal of Botany 32(1): 75–79. 10.1111/j.1756-1051.2013.00306.x

[B10] Möller M (2019) Species Discovery in Time: An Example from Gesneriaceae in China. Guangxi Sciences 26(1): 1–16.

[B11] Möller M Wei YG Wen F Clark JL Weber A (2016) You win some you lose some: Updated generic delineations and classification of Gesneriaceae-implications for the family in China. Guihaia 36: 44–60.

[B12] Ning ZL Pan B Wen F Kang M Zhuang XY (2016) *Primulina yingdeensis*, a new species from Guangdong, China, and *P. rosulata*, a new combination (Gesneriaceae), based on morphological and molecular evidence. Willdenowia 46(3): 399–409. 10.3372/wi.46.46308

[B13] Pan B Xu MZ Tang WX Yang LH (2020) *Primulina zixingensis* (Gesneriaceae), a new species from Hunan, China. Annales Botanici Fennici 57(1–3): 55–59. 10.5735/085.057.0107

[B14] Tropicos (2020) Tropicos.org. Missouri Botanical Garden. http://www.tropicos.org [accessed May 2020]

[B15] Vu PX (2018) Gesneriaceae. In: Tran TH (Ed.) Flora of Vietnam. (Vol. 18). Technology & Science Publishing House, Hanoi.

[B16] Wang YZ Mao RB Liu Y Li JM Dong Y Li ZY Smith JF (2011) Phylogenetic reconstruction of *Chirita* and allies (Gesneriaceae) with taxonomic treatments. Journal of Systematics and Evolution 49(1): 50–64. 10.1111/j.1759-6831.2010.00113.x

[B17] Weber A Middleton DJ Forrest A Kiew R Lim CL Rafidah AR Sontag S Triboun P Wei YG Yao TL Möller M (2011) Molecular systematics and remodeling of *Chirita* and associated genera (Gesneriaceae). Taxon 60(3): 767–790. 10.1002/tax.603012

[B18] Wen F Qin GL Wei YG Liang GY Gao B (2012) Primulina hochiensis var. rosulata (Gesneriaceae)–A new variety at an entrance of a limestone cave from Guangxi, China. Phytotaxa 54: 37–42. 10.11646/phytotaxa.54.1.4

[B19] Wen F Li S Xin ZB Fu LF Cai L Qin JQ Pan B Hong X Pan FZ Wei YG (2019) The Updated Plant List of Gesneriaceae in China against the Background of Newly Chinese Naming Rules. Guangxi Sciences 26(1): 37–63.

[B20] Wen F Wei YG Fu LF Xin ZB Li S Huang ZJ Meng DC (2020) The Checklist of Gesneriaceae in China. http://gccc.gxib.cn/about–46.aspx. [accessed 15 May 2020]

[B21] Yang LH (2018) Species delimitation of *Primulina hochiensis* complex, with some field investigations and taxonomic studies on Gesneriaceae of South China. PhD Thesis, University of Chinese Academy of Sciences, China.

[B22] Yang LH Kong HH Huang JP Kang M (2019) Different species or genetically divergent populations? Integrative species delimitation of the *Primulina hochiensis* complex from isolated karst habitats. Molecular Phylogenetics and Evolution 132: 219–231. 10.1016/j.ympev.2018.12.011 30552965

